# Epidemiology of Emergent Madariaga Encephalitis in a Region with Endemic Venezuelan Equine Encephalitis: Initial Host Studies and Human Cross-Sectional Study in Darien, Panama

**DOI:** 10.1371/journal.pntd.0004554

**Published:** 2016-04-21

**Authors:** Amy Y. Vittor, Blas Armien, Publio Gonzalez, Jean-Paul Carrera, Claudia Dominguez, Anayansi Valderrama, Greg E. Glass, Davis Beltran, Julio Cisneros, Eryu Wang, Alex Castillo, Brechla Moreno, Scott C. Weaver

**Affiliations:** 1 Department of Medicine, University of Florida, Gainesville, Florida, United States of America; 2 Institute for Human Infections and Immunity and Department of Microbiology and Immunology, University of Texas Medical Branch, Galveston, Texas, United States of America; 3 Department of Emerging and Zoonotic Diseases, Gorgas Memorial Institute of Health Studies, Panama City, Panama; 4 Universidad Interamericana de Panama, Panama City, Panama; 5 Department of Research in Virology and Biotechnology, Gorgas Memorial Institute of Health Studies, Panama City, Panama; 6 Department of Medical Entomology, Gorgas Memorial Institute of Health Studies, Panama City, Panama; 7 Department of Geography, University of Florida, Gainesville, Florida, United States of America; The Connecticut Agricultural Experiment Station, UNITED STATES

## Abstract

**Background:**

Neurotropic arboviral infections are an important cause of encephalitis. A zoonotic, vector-borne alphavirus, Madariaga virus (MADV; formerly known as South American eastern equine encephalitis virus), caused its first documented human outbreak in 2010 in Darien, Panama, where the genetically similar Venezuelan equine encephalitis virus (VEEV) is endemic. We report the results of a seroprevalence survey of animals and humans, illustrating contrasting features of MADV and VEEV ecology and epidemiology.

**Methods:**

Small mammals were trapped in 42 sites in Darien, Panama, using Sherman traps, Tomahawk traps, and mist nets for bats. Blood was tested for the presence of neutralizing antibodies to MADV and VEEV. In addition, bird sera collected in 2007 in Chagres, Panama, were tested for MADV and VEEV neutralizing antibodies. Viremia was ascertained by RT-PCR. Human exposure to these two viruses was determined by IgG ELISA, followed by plaque reduction neutralization tests. To identify relevant risk factors for MADV or VEEV exposure, logistic regression analysis was performed, and the most parsimonious model was selected based on the Akaike information criterion.

**Results:**

The animal survey yielded 32 bats (16 species), 556 rodents (12 species), and 20 opossums (4 species). The short-tailed cane mouse (*Zygodontomys brevicauda*) found abundantly in pasture and farms, had the highest MADV seroprevalence (8.3%). For VEEV, the shrub and forest-dwelling long-whiskered rice rat (*Transandinomys bolivaris*) had the highest seroprevalence (19.0%). Viremia was detected in one animal (*Z*. *brevicauda*). Of the 159 bird sera (50 species) tested, none were positive for either virus. In humans (n = 770), neutralizing antibodies to MADV and VEEV were present in 4.8% and 31.5%, respectively. MADV seropositivity was positively associated with cattle ranching, farming, and fishing. Having VEEV antibodies and shrubs near the house diminished risk. Age, forest work, farming and fishing were risk factors for VEEV, while having MADV antibodies, glazed windows, waste pick-up and piped water were protective.

**Conclusion:**

Our findings suggest that the short-tailed cane mouse and the long-whiskered rice rat serve as hosts for MADV and VEEV, respectively. The preferred habitat of these rodent species coincides with areas associated with human infection risk. Our findings also indicate that MADV emerged recently in humans, and that the transmission cycles of these two sympatric alphaviruses differ spatially and in host utilization.

## Author Summary

Arthropod-borne viruses are important causes of encephalitis. In 2010, the first documented human outbreak of the mosquito-borne, zoonotic Madariaga virus (MADV) occurred in the Darien region of Panama. Neither its epidemiology nor its transmission cycle is understood. In this study, the authors searched for possible animal hosts of this virus, and sought to describe its epidemiology. They contrast the findings with those for Venezuelan equine encephalitis virus (VEEV), an endemic, genetically similar virus. *Zygodontomys brevicauda*, the short-tailed cane mouse, had the highest seroprevalence for MADV. This rodent species is most often found in pasture and farm land. Indeed, the risk factors for human MADV exposure were cattle ranching and farming. The animal with highest seroprevalence for VEEV, the long-whiskered rice rat (*Transandinomys bolivaris*), commonly occurs in forest, and the epidemiological risk factors included working in the forest. Farming and fishing were risk factors for exposure to both viruses, and having antibodies to one virus diminished the risk of being positive for the other. Increasing prevalence with age was seen for VEEV, confirming that VEEV is endemic in the region. This association was absent for MADV, suggesting that this virus emerged recently to infect humans.

## Introduction

Madariaga (MADV) and Venezuelan equine encephalitis viruses (VEEV) are mosquito-borne, single-stranded positive sense RNA viruses (family *Togaviridae*, genus *Alphavirus)*. Both circulate nearly throughout the Americas. In Central and South America, VEEV gives rise to a spectrum of disease in humans, ranging from undifferentiated fevers to fatal encephalitis and hemorrhage. However, unlike its northern counterpart, Madariaga (MADV, formerly known as South American EEEV) was not associated with human outbreaks prior to 2010, when the first known human MADV outbreak was reported from Darien, Panama [1]. During this outbreak, MADV and VEEV co-circulated, with over 100 suspected cases and 19 hospitalizations for encephalitis. MADV was confirmed in 13 cases, VEEV in 11, and one case of dual infection was detected.

There is significant divergence between North American eastern equine encephalitis (NA EEEV) and Madariaga virus [2], raising the potential for differences in transmission cycles and virulence [3]. Studies in Peru revealed that, while isolation of MADV from mosquitoes known to feed on humans was common [4], no MADV was isolated in acutely febrile patients, and overall seroprevalence was very low [5]. Dietz et al. [6] investigated an equine outbreak of MADV in Panama in 1973, and found that none of the 1700 humans surveyed in the same region was seropositive for MADV. Phylogenetic analysis of MADV strains isolated in the 2010 outbreak revealed that the circulating virus was very similar to the 1984 and 1986 Panama isolates associated with equine outbreaks, and thus was not a recently imported strain. The mechanism of enzootic circulation and the drivers of emergence of MADV as a human pathogen remain unknown.

By comparison, explosive VEEV epidemics and epizootics involving equine amplification have resulted in up to 100,000 human cases and thousands of equine fatalities in Latin America [7]. However, VEE resulting from enzootic strain spillover is underdiagnosed, due to the lack of readily available diagnostic tools and the extensive overlap in signs and symptoms with dengue and other acute febrile infectious diseases. Up to 10% of clinical dengue cases in Neotropical regions in Latin America may actually be VEEV [7]. *Culex* species of the subgenus *Melanoconion* have been incriminated as enzootic vectors in Latin America for MADV and VEEV [8]. Both viruses are thought to be maintained in stable enzootic cycles in between epizootics/epidemics. The evidence to date suggests that rodents are the principle hosts in the VEEV enzootic cycles [9], while definitive evidence of enzootic hosts is lacking for MADV in Latin America.

Elucidating the hosts of enzootic MADV is essential to understanding transmission dynamics, especially following the emergence of MADV as a human pathogen. A good amplification host is susceptible to infection, develops high titers of prolonged viremia, and is also host to an arthropod vector that is competent for transmission [10]. Serology and detection of viremia serve to initially screen potential amplifying hosts, identifying currently infected species or those with a history of exposure to the virus. Prior studies of MADV infection in wildlife have found members of the rodent genus *Oryzomys*, the common opossum (*Didelphis marsupialis*), and several bird species to be viremic [6,11–15]. Too few reptile species and specimens have been examined to draw conclusions [13], but there is intriguing evidence of sustained NA EEEV viremia in snakes [16]. A host competence study involving experimental infections of cotton rats (*Sigmodon hispidus berlandieri*) and house sparrows (*Passer domesticus*) demonstrated that juvenile rats developed higher levels of MADV viremia than adults, while the house sparrows developed higher levels of viremia with NA EEE than MADV [10]. The biting behavior of the proven VEEV and probable MADV mosquito vector (*Culex (Mel*.*) taeniopus*) is thought to be fairly catholic in some studies [17], though a proclivity for rodent feeding was seen in others [8,18].

In 2012, we conducted a cross-sectional serological survey in select villages in Darien, Panama, a region involved in the 2010 encephalitis outbreak. Our goal was to identify risk factors and ecological features associated with MADV and VEEV exposure in small mammals and humans to better understand the transmission dynamics of the two viruses. Here, we describe the results of serosurvey and risk factors associated with MADV and VEEV seropositivity, which provides insights into similarities and differences of their transmission and host susceptibilities to these two viruses.

## Methods

### Study area

**Fig 1:**
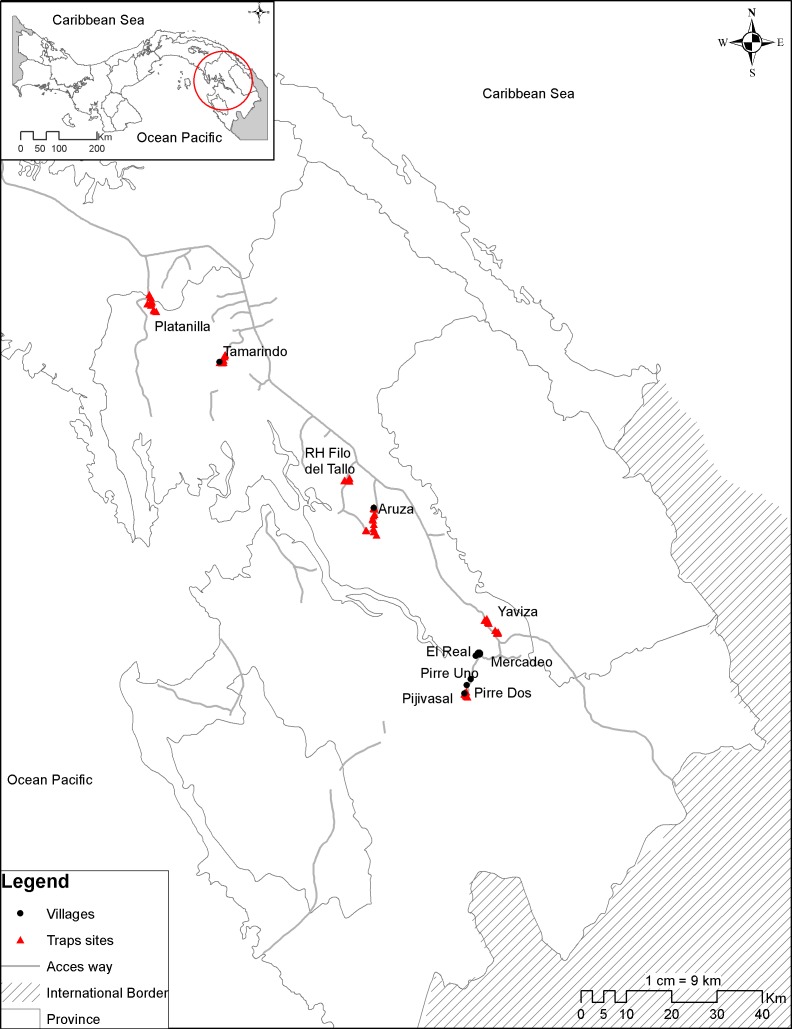
Map of the study region.

The study took place in the province of Darien, Panama (8.42 N 78.15W) (Fig 1). Darien is the easternmost province of Panama, bordered by the Pacific Ocean and Colombia. It is home to the Parque Nacional del Darien, also known as the Darien Gap. The largest national park in Central America and a UNESCO Biosphere Reserve, it is biologically, culturally, and ethnically diverse. The Pan-American Highway ends at the town of Yaviza, rendering much of the park inaccessible. Yearly rainfall ranges between 1,800 and 4,500mm, with drier areas located in the central and southern gulf and wetter areas situated along the inland and Caribbean mountains. The mean maximum temperature is 28°C during the rainy season and 31°C in the dry season[19]. The animal trapping effort took place from January 2012 through December 2012 in six locations: 1) Tamarindo, 2) Aruza, 3) Platanilla, 4) Filo del Tallo, 5) Yaviza, and 6) Pijivasal. The epidemiological study included five village clusters: 1) Tamarindo, 2) Aruza, 3) El Real de Santa Maria, 4) Mercadeo, and 5) Pijivasal, Pirre 1 and Pirre 2. These animal and human serosurveillance sites were chosen based on the Panamanian Ministry of Health reports on the occurrence of encephalitis cases between the years 2002 and 2012.

Tamarindo, Aruza and Platanilla are agricultural villages populated by mestizos engaged in rice and maize farming, and cattle ranching. These towns are near the Pan-American Highway. Filo del Tallo is a small watershed forest reserve that spans 240km^2^. Yaviza lies at the end of the highway, and has an urban center surrounded by agricultural fields. El Real de Santa Maria is a relatively densely populated town on the rivers Tuira and El Real. It is inhabited primarily by Afro-Panamanian people and was settled nearly 400 years ago. Lying beyond the Pan-American Highway, it is only accessible by boat. People in this town include professionals, retirees, and to a lesser degree, farmers. Mercadeo lies upstream from El Real de Santa Maria on the river Tuira. Further upstream on the river Pirre is the small village Pijivasal. There is a short road connecting El Real de Santa Maria and Pijivasal, along which additional small towns (Pirre 1 & 2) are located. Primary sources of income in these villages are farming, with some cattle ranching activity in Pirre 1 and Pirre 2. Mercadeo and Pijivasal are inhabited mainly of members of the indigenous Embera-Wounaan tribe.

### Ethics

The study was approved by the Gorgas Memorial Institute Ethics Committee (IRB # 047/CNBI/ICGES/11), the University of Texas Medical Branch (IRB # 95–111), and the University of Florida Institutional Review Board (IRB # 201500292). Study participation was voluntary, and written informed consent was obtained from adults and parents or guardians for children age 2 and older. In addition, assent was obtained from children ages 7 to 12, and informed consent was obtained from children ages 13 to 17. The capture, use, and euthanization of wild animals was evaluated and approved by the Institutional Animal Care and Use Committee of the Gorgas Memorial Institute for Health Studies (# 012/05 CIUCAL/ICGES, 2010) and the Panamanian National Environment Authority (SC/A-32-10, 2010, ANAM) using the criteria established in the "International Guiding Principles for Biomedical Research Involving Animals developed by the Council for International Organizations of Medical Sciences (CIOMIS). The study was in accordance with Law No. 23 of January 15, 1997 (Animal Welfare Assurance) of República de Panamá.

### Study design

This study was part of a larger initiative to define prevalence of zoonotic diseases in Panama, undertaken by the Department of Emerging Zoonotic Diseases of the Gorgas Memorial Institute. Field expeditions were undertaken for the capture of wild animals and the collection of human sera and questionnaires. A cross-sectional survey was carried out in each village. If villagers could not be located (usually because they were away at work or traveling), two additional attempts were made to contact residents. In Tamarindo, 59 of 86 (69%) households were surveyed; in Aruza, 47 of 60 (78%) were surveyed; in Mercadeo, 18 of 32 (56%) were surveyed; in Pirre 1 & 2, 9 of 15 (60%) were surveyed; and in Pijivasal 9 or 14 (64%) were surveyed. Due to El Real’s large size, every third household in El Real was surveyed (82 or 178 households, 46%). All consenting household members age two years and older were included. In most instances, lack of participation was due to the inability to locate the household residents. The refusal rates were 3% (n = 6) for Tamarindo, 2% (n = 2) for Aruza, 5% (n = 6) for Mercadeo, 0% in Pirre 1 & 2, 0% in Pijivasal, and 1% (n = 3) in El Real.

#### Rodent and opossum trapping

Between January and December, 2012, 4 to 8 sites were set up in each of the six study areas (42 sites total). In each site, 100 rodent traps (Sherman Traps, Inc., Tallahassee, FL) were placed at 10m intervals in a grid[20]. These were baited with a mixture of rice, corn, sorghum, and peanut butter. Larger traps (Tomahawk Live Trap, LLC, Hazelhurst, WI) were placed at each corner to capture opossums (4 total in a grid). These traps were baited with tuna. At each location, the trap sites were sampled simultaneously for one to five consecutive nights. Trapped animals were euthanized using halothane inhalation. The animals were identified, sexed and aged. Blood was obtained by cardiac puncture, and organs were harvested. Species were identified using keys by Reid (2009)[21]. All of the samples were immediately frozen and kept in liquid nitrogen. Once brought to the Gorgas Memorial Institute, the samples will be stored at -80°C.

#### Bat trapping

Bats were trapped in Yaviza, Platanilla, Aruza, and Tamarindo between January and October, 2012. In each location, two to four mist nets were strung near houses from dusk to 11pm for two consecutive nights. Trapped bats were handled in the same fashion as the rodents and opossums (euthanized, identified, aged, sexed, and whole blood obtained by cardiac puncture).

#### Bird sera

During a prior sampling effort, birds were trapped in 2007 in Chagres, north of Darien. Birds were captured using mist nets during four consecutive days per month between 6am and 6pm. A volume of 0.1ml to 0.3ml of blood was drawn from small birds (6gm– 76gm) and 0.3ml to 1.0ml from larger birds (>76gm). Sera from these birds stored at -80°C were tested, although volumes were insufficient for RT-PCR.

### Serosurveillance in humans

Surveys were conducted between January and December, 2012. Participants 2 years of age or older were eligible for inclusion. Each participant was interviewed using a standardized questionnaire pertaining to travel history, occupation, activities, livestock and crop holdings (S5 Table). Household-level information was obtained from the head of the household, and observable household-level data (e.g. house structure) were recorded directly by the interviewer. Trained phlebotomists collected 10 ml of blood (3ml for children 2–8 years old) by peripheral venipuncture using standard aseptic technique. The samples were processed on-site within 12 hours by centrifugation to separate serum, then stored in liquid nitrogen, and transported to the Gorgas Memorial Institute for testing.

### Laboratory assays

All human serum samples were screened in duplicate for IgG antibodies to MADV and VEEV antigen by enzyme-linked immunosorbent assays (ELISA)[22], and if indeterminate or positive, infection was confirmed by a plaque-reduction neutralization test (PRNT)[23]. The neutralizing antibody titer was determined as the reciprocal of the highest dilution that reduced plaque count by ≥80% (PRNT_80_). PRNT is specific for MADV and VEEV, and cross-reactivity for these two viruses has not been reported [5]. For the ELISA, sucrose-acetone antigens were prepared from EEEV- (prepared by Dr. Robert Shope at the Yale Arbovirus Research Unit in August 1989) and VEEV- (strain 78V-3531) infected mouse brain. For the PRNT, we used chimeric SINV/MADV (derived from Brazilian MADV strain BeAn436087 [24], shown to be an accurate surrogate for MADV in these assays [23] and TC83, an attenuated vaccine strain of VEEV closely related to subtype ID strains that circulate in Panama [25]. All animal blood samples were tested for MADV and VEEV using PRNT. Viral isolation in Vero E6 cells was also attempted for bird sera.

Detection of Alphavirus nucleic acid was accomplished by RT-PCR using universal alphavirus primers [26]. RNA was extracted from homogenized animal spleens using the QIAamp Viral RNA Mini kit (Qiagen, Valencia, CA, USA). RT-PCR was then performed in a 50μl reaction volume using the Titan One Tube RT-PCR system according to manufacturer’s protocol (Roche Life Science, Indianapolis, IN, USA). If a 481bp amplicon was present, the amplicon was eluted from the agarose gel, purified (QIAquick PCR purification kit, Qiagen) and sequenced using an Applied Biosystems 310 Genetic Analyzer (Foster City, CA) according to the manufacturer’s protocols. The viral sequences were identified using BLAST searches of the GenBank database.

### Statistical methods

#### Animal data

The abundance of rodent species was tabulated. For each species, MADV and VEEV seroprevalence rates were calculated by dividing the number of seropositive animals (PRNT_80_≥40) by the total number collected. Descriptive measures such as means and their confidence intervals are presented.

#### Epidemiological data

Two sets of analyses were conducted for each of the two viruses. The outcome variable was defined as absence or presence of MADV or VEEV antibodies as determined by PRNT_80_≥20. Independent variables included: age, sex, duration of occupancy, migration history, occupation, activities (farming, poultry husbandry, cattle ranching, hog farming, hunting, fishing, bathing and washing in rivers), types of crops held, type of livestock held, house construction, assessment of waste and shrubs surrounding home, waste management, water supply, and socioeconomic indicators. Univariate logistic regression with clustered robust standard errors (to account for the correlation of household members) was used to identify risk factors for MADV and VEEV exposure separately. Data reduction using exploratory factor analysis was explored, but ultimately not used since resultant variables were not superior to the original variables. Multivariate analysis was undertaken by adding variables from the univariate analysis with p<0.25 using purposeful selection as described by Hosmer and Lemeshow[27]. Only variables that remained significant were included in the final models. Interaction variables between all variables identified as significant in the univariate analysis were examined (S5 Table), and marginal effects of significant interaction terms were determined. Model diagnostics were performed to check for model specification errors, multicollinearity and influential observations, and model selection was based on the Akaika Information Criterion (AIC). Model fit was ascertained using the Hosmer-Lemeshow goodness of fit statistic. Data were analyzed in Stata Statistical Software release 12.1 (StataCorp, College Station, TX).

## Results

### Animal survey

A total of 556 rodents (12 species) was trapped in 11,500 trap-nights and 20 opossums (4 species) in 505 trap nights. In addition, 32 bats (16 species) were trapped and sera from 159 birds (50 species) were tested. Two opossum species and four rodent species were positive for VEEV antibodies (Tables 1 and 2). Four rodent species and two bat species were positive for MADV antibodies (Tables 2 and 3). None of the bird sera tested positive for exposure to either virus by PRNT (Table 4) or by viral isolation. Only one specimen (*Zygodontomys brevicauda*) was positive for alphavirus RNA by RT-PCR. The amplicon was identified as enzootic VEEV (subtype ID) by sequencing. The complete list of PRNT titers can be found in S1 Table.

**Table 1:** MADV and VEEV seroprevalence* in animals by species–opossums.

Family	*Species*	Common Name	N	MADV (%)	VEEV (%)
Didelphidae	*Caluromys derbianus*	Derby’s woolly opossum	1	0	0
	*Marmosa robinsoni*	Robinson’s mouse opossum	5	0	2 (40.0)
	*Didelphis marsupialis*	Common opossum	13	0	3 (23.1)
	*Metachirus nudicaudatus*	Brown four-eyed opossum	1	0	0

*Based on PRNT results

**Table 2:** Seroprevalence* by species–rodents.

Family	*Species*	Common Name	N	MADV (%)	VEEV (%)
Cricetidae	*Transandinomys bolivaris*	Long-whiskered rice rat	164	5 (3.1)	31 (19.0)
	*Zygodontomys brevicauda*	Short-tailed cane mouse	229	19 (8.3)	17 (7.5)
	*Sigmodon hirsutus*	Southern cotton rat	11	0	0
	*Reithrodontomys fulvescens*	Fulvous harvest mouse	1	0	0
	*Oligoryzomys fulvescens*	Northern pygmy rice rat	1	Nd†	Nd
	*Melanomys caliginosus*	Dusky rice rat	1	0	1
	*Oecomys sp*.	Oecomys	1	0	0
Heteromyidae	*Heteromys desmarestianus*	Desmaret’s spiny pocket mouse	1	0	0
	*Heteromys australis*	Southern spiny pocket mouse	2	0	0
Echimyidae	*Proechimys semispinosus*	Tome’s spiny-rat	67	1 (1.5)	3 (4.5)
Muridae	*Rattus rattus*	Black rat	76	3 (3.9)	0
	*Rattus norvegicus*	Brown rat	2	0	0

*Based on PRNT results

† Not done

**Table 3:** Seroprevalence* by species–bats.

Family	*Species*	Common Name	N	MADV (%)	VEEV (%)
Phyllostomidae	*Carollia castanea*	Chestnut short-tailed bat	1	0	0
	*Carollia perspicillata*	Seba’s short-tailed bat	9	1 (11.1)	0
	*Phyllostomus discolor*	Pale spear-nosed bat	4	1 (25.0)	0
	*Phyllostomus hastatus*	Greater spear-nosed bat	3	0	0
	*Phyllostomus sp*.	Spear-nosed bat	1	0	0
	*Uroderma bilobatum*	Tent-making bat	3	0	0
	*Artibeus jamaicensis*	Mexican fruit bat	1	0	0
	*Mimon crenolatum*	Striped hairy-nosed bat	1	0	0
	*Carollia subrufa*	Gray short-tailed bat	1	0	0
	*Artibeus lituratus*	Great fruit-eating bat	1	0	0
	*Vampyressa nymphaea*	Striped yellow-eared bat	2	Nd†	Nd
	*Glyphonycteris sylvestris*	Tricolored big-eared bat	1	Nd	Nd
Mormoopidae	*Pteronotus parnellii*	Parnell’s mustached bat	1	0	0
Vespertilionidae	*Eptesicus brasiliensis*	Brazilian brown bat	1	0	0
	*Eptesicus furinalis*	Argentine brown bat	1	0	0
	*Myotis sp*.	Mouse-eared bat	1	Nd	Nd

*Based on PRNT results

† Not done

**Table 4:** Seroprevalence* by species–birds.

Family	*Species*	Common Name	N	EEEV (%)	VEEV (%)
Pipridae	*Chiroxiphia lanceolata*	Lance-tailed manakin	5	0	0
	*Manacus vitellinus*	Golden-collared manakin	5	0	0
	*Pipra mentalis*	Red-capped manakin	5	0	0
Formicariidae	*Formicarius analis*	Black-faced antthrush	5	0	0
Parulidae	*Parkesia noveboracensis*	Northern waterthrush	5	0	0
	*Geothlypis formosa*	Kentucky warbler	1	0	0
	*Helmitheros vermivorum*	Worm-eating warbler	1	0	0
Thamnophilidae	*Cercomacra tyrannina*	Dusky antbird	5	0	0
	*Hylophylax naevioides*	Spotted antbird	5	0	0
	*Thamnophilus atrinucha*	Black-crowned antshrike	4	0	0
	*Myrmeciza longipes*	White-bellied antbird	1	0	0
Troglodytidae	*Cyphorhinus phaeocephalus*	Song wren	6	0	0
	*Thryothorus rufalbus*	Rufous-and-white wren	3	0	0
	*Thryothorus rutilus*	Rufous-breasted wren	1	0	0
	*Pheugopedius fasciatoventris*	Black-bellied wren	5	0	0
Turdidae	*Turdus grayi*	Clay-colored thrush	5	0	0
	*Catharus ustulatus*	Swainson’s thrush	3	0	0
	*Catharus minimus*	Grey-cheeked thrush	4	0	0
Tyrannidae	*Attila spadiceus*	Bright-rumped Attila	1	0	0
	*Tolmomyias assimilis*	Yellow-margined flatbill	2	0	0
	*Onychorhynchus coronatus*	Royal flycatcher	4	0	0
	*Schiffornis turdinus*	Schiffornis	5	0	0
	*Rhynchocyclus olivaceus*	Olivaceous flatbill	1	0	0
	*Mionectes oleaginous*	Ochre-bellied flycatcher	6	0	0
	*Terenotriccus erythrurus*	Ruddy-tailed flycatcher	5	0	0
	*Myiobius atricaudus*	Black-tailed flycatcher	1	0	0
	*Empidonax virescens*	Acadian flycatcher	2	0	0
Bucconidae	*Malacoptila panamensis*	White-whiskered puffbird	1	0	0
Momotidae	*Momotus momota*	Blue-crowned motmot	4	0	0
Cerylidae	*Chloroceryle aenea*	American pygmy kingfisher	1	0	0
Polioptilidae	*Ramphocaenus melanurus*	Long-billed gnatwren	1	0	0
Emberizidae	*Arremon aurantiirostris*	Orange-billed sparrow	4	0	0
Caprimulgidae	*Nyctidromus albicollis*	Pauraque	1	0	0
Thraupidae	*Rhodinocichla rosea*	Rosy thrush-tanager	5	0	0
	*Tangara inornata*	Plain-colored tanager	2	0	0
	*Saltator maximus*	Buff-throated saltator	2	0	0
	*Tachyphonus luctuosus*	White-shouldered tanager	2	0	0
	*Sporophila Americana*	Wing-barred seedeater	1	0	0
	*Oryzoborus funereus*	Thick-billed finch	2	0	0
Furnariidae	*Xiphorhunchus susurrans*	Coca woodcreeper	6	0	0
	*Xenops minutus*	Plain xenops	5	0	0
	*Sittasomus griseicapillus*	Olivaceous woodcreeper	3	0	0
	*Sclerurus guatemalensis*	Scaly-throated leaftosser	3	0	0
	*Dendrocincla fuliginosa*	Plain-brown woodcreeper	1	0	0
Columbidae	*Leptotila cassini*	Grey-chested dove	5	0	0
	*Leptotila verreauxi*	White-tipped dove	1	0	0
	*Geotrygon montana*	Ruddy quail-dove	2	0	0
Cardinalidae	*Piranga rubra*	Summer tanager	1	0	0
	*Habia fuscicauda*	Red-throated ant tanager	5	0	0
	*Cyanocompsa cyanoides*	Blue-black grosbeak	5	0	0

*Based on PRNT results

One of four *Phyllostomus discolor*, and one of nine *Carollia perspicillata* bats had MADV antibodies. Among the rodents, the short-tailed cane mouse, *Zygodontomys brevicauda*, had the highest rate of MADV seroprevalence [n = 229; 8.3%, 95% CI (4.8–12.2)] followed by the black rat (*Rattus rattus*) [n = 76; 3.9%, 95% CI (-0.5–8.0)], the long-whiskered rice rat (*Transandinomys bolivaris*) [n = 164; 3.1%, 95% CI (0.4–5.7)], and Tome’s spiny rat (*Proechimys semispinosus*) [n = 67; 1.5%, 95% CI(-1.5–4.5)].

Four of thirteen common opossums (*Didelphis marsupialis*) and two of five Robinson’s mouse opossums (*Marmosa robinsoni*) were VEEV-seropositive. The single dusky rice rat (*Melanomys caliginosus*) collected was seropositive for VEEV. The long-whiskered rice rat had the highest VEEV seroprevalence (19.0%, 95% CI(12.9–25.1)), followed by the short-tailed cane mouse (7.5%, 95% CI(3.7–10.5)), and Tome’s spiny rat (4.5%, 95% CI(-0.6–9.7)). Three specimen of *Z*. *brevicauda* and *T*. *bolivaris* each (6 total) were seropositive for both viruses.

The short-tailed cane mouse was found predominantly in pasture and cultivated land, while the black rat was captured mainly in populated areas (in towns, villages). The long-whiskered rice rat and Tome’s spiny rat were found mostly in mature and secondary forests, respectively.

### Serosurveillance in humans

A total of 787 people (ages 2–88) was enrolled and serological results were obtained for 770 (97.8%). For 17 individuals, questionnaires, but not blood, were obtained and these were removed from the analysis. The proportions of enrolled men and women were approximately equal, and children and young adults predominated (Table 5). The vast majority (96.2%) of participants had lived in their house for more than a year, and most (68.4%) had never lived in a different province. There was substantial geographic variation in the distribution of age, activities, livestock, house structure, and infrastructure (Table 5). Compared to those in the other communities, the study participants in Aruza had significantly greater exposure to cattle and pasture, while those in Mercadeo and Pijivasal/Pirre 1 & 2 engaged in more hunting and fishing. People in El Real de Santa Maria reported significantly less participation in agricultural activities (farming, hunting, fishing, cattle ranching, owning livestock). House construction and infrastructure varied by community as well. Houses generally had wooden walls, with some use (25%) of corrugated metal for walls in Mercadeo. Dirt floors predominated in Tamarindo, while other communities used wood or cement. Roofs in Tamarindo and Aruza were mostly palm thatch; in El Real de Santa Maria, corrugated metal was used almost exclusively for roofs, and in Mercadeo and Pijivasal/Pirre 1 & 2 thatch and corrugated metal were equally prevalent. El Real de Santa Maria was the only community where windows were glazed (33.1%), and was also the only community with municipal waste pick-ups. In other communities, waste was buried, burned, or taken to a local dump. Most participants had access to piped water, though this was less so for those living in Mercadeo (44.7%), and entirely absent for the inhabitants of Pijivasal/Pirre 1 & 2 (0%) where the river or rain was a common source of drinking water.

**Table 5:** Summary of study participant characteristics, agricultural practices, and housing by village, Darien, Panama.

Variable	Village									
	Tamarindo (n = 176)		Aruza (n = 167)		El Real (n = 250)		Mercadeo (n = 103)		Pijivasal/ Pirre 1&2 (n = 74)	
	n	%	n	%	n	%	n	%	n	%
**Age quintiles**										
2–9	27	(15.3)	36	(21.6)	60	(24.0)	29	(28.2)	20	(27.0)
10–15	26	(14.8)	25	(15.0)	45	(18.0)	30	(29.1)	14	(18.9)
16–29	43	(24.4)	42	(25.2)	36	(14.4)	17	(16.5)	22	(29.7)
30–48	50	(28.4)	36	(21.6)	40	(16.0)	18	(17.5)	8	(10.8)
≥49	30	(17.1)	28	(16.8)	69	(27.6)	9	(8.7)	10	(13.5)
Male	94	(53.4)	89	(53.3)	114	(45.6)	51	(49.5)	37	(50.0)
Female	82	(46.6)	78	(46.7)	136	(54.4)	52	(50.5)	37	(50.0)
Migrants^1^	88	(50.0)	69	(41.3)	61	(24.3)	11	(10.7)	16	(21.6)
**Activities**										
Farming	57	(32.4)	48	(28.7)	19	(7.6)	14	(13.6)	15	(20.3)
Fishing	13	(7.4)	21	(12.6)	19	(7.6)	16	(15.5)	15	(20.3)
**Land use**										
Pasture	9	(5.1)	29	(17.4)	3	(1.2)	1	(1.0)	5	(6.8)
Rice	52	(29.6)	51	(30.5)	27	(10.8)	31	(30.1)	27	(36.5)
Maiz	59	(33.5)	65	(38.9)	24	(9.6)	27	(26.2)	23	(31.1)
**Livestock**										
Poultry	152	(86.4)	117	(70.1)	105	(42.0)	79	(76.7)	69	(93.2)
Pig	100	(56.8)	83	(49.7)	2	(0.8)	0	(0)	15	(20.3)
Cattle	48	(27.3)	83	(49.7)	0	(0)	0	(0)	5	(6.8)
Horse	115	(65.3)	104	(62.3)	6	(2.4)	13.6	(13.6)	20	(27.0)
**House features**										
Wooden floors^2^	19	(10.8)	66	(39.5)	114	(45.4)	103	(100)	70	(94.6)
Wooden walls^3^	165	(93.8)	154	(92.2)	188	(75.2)	73	(70.9)	60	(81.1)
Glass windows^4^	7	(4.0)	3	(1.8)	83	(33.2)	0	(0)	0	(0)
Corrugated metal roof^5^	95	(54.0)	122	(73.1)	246	(98.0)	78	(75.7)	68	(91.9)
Municipal waste^6^	0	(0)	0	(0)	176	(70.1)	6	(5.8)	0	(0)
Piped water^7^	165	(93.8)	111	(66.5)	235	(94.0)	46	(44.7)	0	(0)
Electricity	141	(80.1)	48	(28.7)	237	(94.8)	74	(71.8)	70	(94.6)

^1^ Migrants from a different province, mostly Herrera and Los Santos

^2^ Other floor types were: cement, tile, dirt

^3^ Other wall types were: cement, corrugated metal, mud

^4^ Other window types were: open windows, wooden shutters

^5^ Other roofing materials were: thatch, tile

^6^ Other waste disposal methods were: burying, burning, local garbage heap, disposing of waste into river

^7^ Other means of acquiring water were: wells, river, rain water collection

The crude seroprevalences of MADV (4.81%; n = 770) and VEEV (31.34%; n = 769) were significantly different. Ten participants had dual exposure. The towns with the highest MADV seroprevalence were not those with the highest prevalence of VEEV. Aruza had an MADV seroprevalence of 16.2%, while the other four communities had much lower rates (<4%, p<0.001). In contrast, VEEV seroprevalence was highest in Pijivasal/Pirre 1 & 2 (78.1%), while Mercadeo, Aruza and El Real de Santa Maria had rates that were a third to one half that seen in Pijivasal/Pirre 1 & 2 (44.7%, 32.9% and 27.2%, respectively), and Tamarindo had a VEEV seroprevalence of only 8.5% (p<0.001). The complete list of PRNT titers can be found in S2 Table.

### MADV risk factors

**Fig 2:**
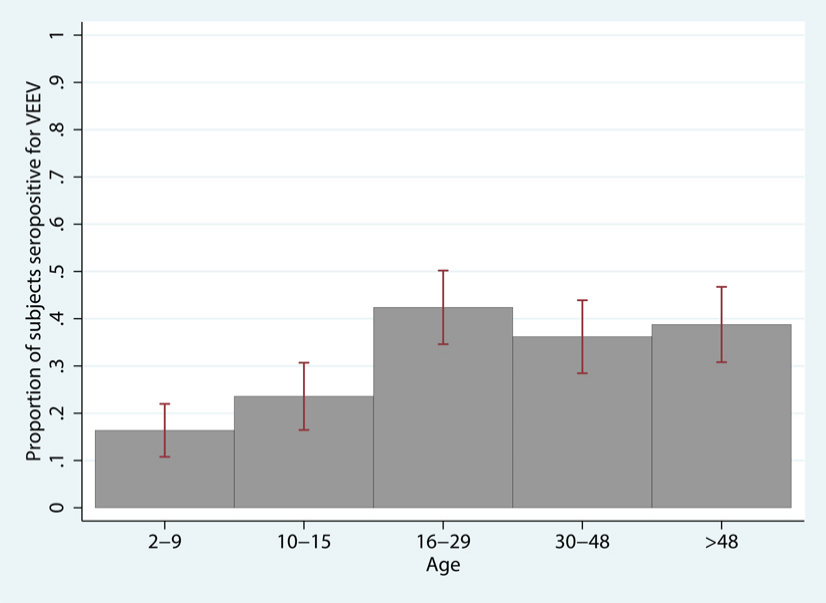
Age structure of MADV seroprevalence. Table displays odds ratios, 95% confidence interval, and p-value for univariate logistic regression of MADV seropositivity with each unit increase in age (unit = one year).

In the univariate analysis, there were significant positive associations for the presence of MADV antibodies with pasture and cattle ranching (owning cattle and/or horses, being a cattle rancher, walking through/playing in/working in pasture, owning pasture land), farming (being a farmer, growing crops such as rice, watermelon, and cassava root, walking through or playing in crop fields), fishing, and particular house features (mud floors, corrugated metal walls, thatched roofs, presence of a well). Significant protective factors (inversely correlated with MADV seropositivity) included having shrubs within 10m of the house and having municipal waste management (versus burning or burying waste, throwing in into the river, or bringing it to a local dump). Data reduction (principal components analysis) did not yield results that were more informative than the original variables, and was ultimately not incorporated. Multivariate analysis revealed that nearby shrubs were protective for MADV, and exposure to farming and crop fields, cattle ranching, and fishing remained significant risk factors, even when controlling for community (ie. unmeasured community level factors) (Table 6). An interaction term for the presence of VEEV antibodies (as determined by PRNT) by community was found to be significant. Having VEEV antibodies was negatively associated with MADV seropositivity in Aruza, but this association was reversed in the other communities. S3 Table illustrates the marginal effects of VEEV antibodies on the outcome, MADV seropositivity, by community when holding all other variables in the model at their mean value. In Aruza, the probability of being MADV seropositive is decreased by 12.6% (p = 0.001) in those who have concurrent VEEV antibodies compared to those who do not, while the probability is increased by 1.7% in those with VEEV antibodies vs. those without VEEV antibodies in the other communities (not significant). MADV seroprevalence was not associated with age, suggesting a lack of long-term endemicity (Fig 2). The model fit the data well (Hosmer-Lemeshow goodness-of-fit test *χ*^2^ = 9.1, *p* = 0.24), and the model variables demonstrated no multicollinearity.

**Table 6:** Multivariate logistic regression model of MADV seroprevalence risk factors.*

Risk Factor	N	% MADV Ab positive	Adjusted OR	OR 95% CI	P>|z|
**Activities**					
Cattle ranching					
0–19 hrs/week	753	4.4	*Ref*		
≥20 hrs/week	19	21.1	2.4	(1.0–5.6)	0.041
Farm exposure					
No	372	1.9	*Ref*		
Yes	398	7.5	3.1	(1.3–7.4)	0.013
Fishing					
0–9 hrs/week	775	4.5	*Ref*		
≥10 hrs/week	10	20.0	8.2	(1.3–52.9)	0.027
**House features**					
Shrub within 10m of house					
No	542	6.3	*Ref*		
Yes	228	1.3	0.2	(0.1–0.6)	0.003
**Host factors**					
VEEV Ab absent, site ≠ Aruza†	415		*Ref*		
VEEV Ab present, site ≠ Aruza†	186		4.2	(1.1–16.9)	0.040
VEEV Ab absent, site = Aruza	112		32.2	(9.3–111.3)	<0.001
VEEV Ab present, site = Aruza	55		0.03	(0.0–0.2)	<0.001

*Adjusted for sex (*p* = 1.0) and age (*p* = 0.68), based on PRNT results

†This refers to sites other than Aruza, namely Tamarindo, El Real, Mercadeo, Pirre 1 & 2 and Pijivasal

Likelihood-ratio (LR) = 87.60; *df* = 9; *p* <0.001

Hosmer-Lemeshow *χ*^2^ = 3.99; *df* = 8; *p* = 0.86

### VEEV risk factors

**Fig 3:**
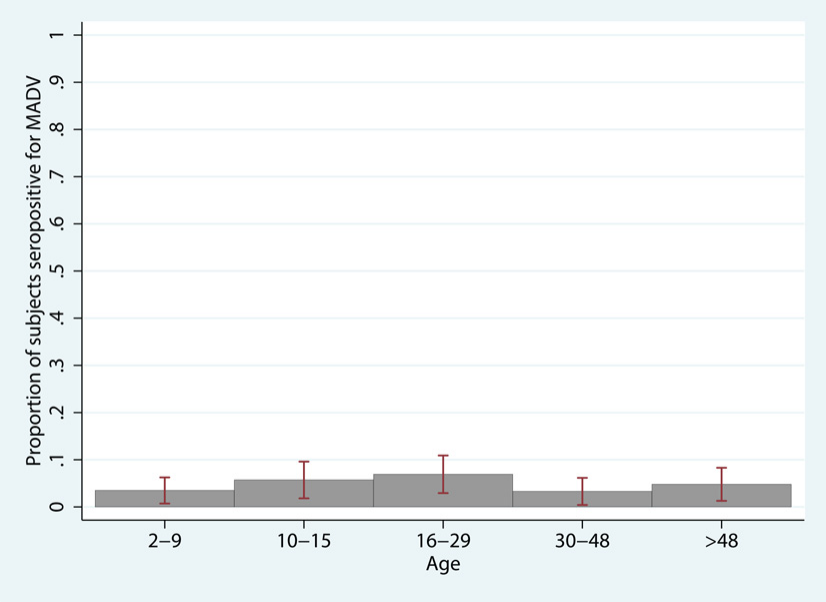
Age structure of VEEV seroprevalence. Table displays odds ratios, 95% confidence interval, and p-value for univariate logistic regression of VEEV seropositivity with each unit increase in age (unit = one year).

VEEV seroprevalence increased with age (Fig 3), consistent with stable, endemic exposure. Seroprevalence in children ages 2 to 5 was 11.1% (95% CI 4.1–18.1%), doubled by age 10 to 23.1% (95% CI 1.6–30.8) and stabilized in individuals > 15 y.o. age categories (39.2%, 95% CI 34.7–43.7). In univariate analysis, VEEV was associated with farming (being a farmer, growing crops such as maize, rice, sugarcane, watermelon, and yams, walking through or playing in crop fields), being in the forest (hunting, logging), activities involving rivers (bathing and/or washing clothes in the river, fishing), cattle ranching and owning/working in pasture, and variables associated with the home (being a homemaker, having wooden floors, tile or straw roof, nearby shrubs). Protective factors included having windows with glass panes (versus open windows or windows with wooden shutters), having cement floors, owning pigs, having municipal waste management, and piped water. Farming, working in the forest, fishing, having glazed windows, municipal waste pick-up, and piped water remained significantly associated with VEEV seropositivity in multivariate analysis, even when controlling for age and community (Table 7). As in the model for MADV above, an interaction term for the presence of MADV antibodies by community was found to be significant. S4 Table illustrates the marginal effects of MADV antibodies on VEEV seropositivity, holding all other model variables at their mean value. In Aruza, the probability of being VEEV seropositive is decreased by 15.6% (p<0.001) in those who have concurrent MADV antibodies compared to those who do not, while the probability of being VEEV seropositive is increased by 40.8% in those with MADV- vs. those without MADV- antibodies in the other communities (p = 0.022). With a p-value of 0.12 (*χ*^2^ = 12.81) in the Hosmer-Lemeshow’s goodness-of-fit test, we conclude that the model fit the data well. The model variables were checked for multicollinearity, and none was observed.

**Table 7:** Multivariate logistic regression model of VEEV seroprevalence risk factors.*

Risk Factor	N	% VEEV Ab positive	Adjusted OR	OR 95% CI	P>|z|
**Activities**					
Farming					
No	498	24.3	*Ref*		
Yes	271	44.3	2.4	(1.6–3.5)	<0.001
Working in the forest					
No	731	29.8	*Ref*		
Yes	38	60.5	3.8	(1.4–10.6)	0.010
Fishing					
0–8 hrs/week	758	30.5	*Ref*		
≥9 hrs/week	11	90.9	10.8	(2.5–46.0)	0.001
**House features**					
Glazed windows					
No	720	32.8	*Ref*		
Yes	49	10.2	0.3	(0.1–0.9)	0.027
Municipal waste pick up					
No	587	34.4	*Ref*		
Yes	182	21.4	0.5	(0.3–0.9)	0.031
Piped water					
No	212	55.7	*Ref*		
Yes	557	22.1	0.5	(0.3–0.9)	0.019
**Host factors**					
Age			1.02	(1.00–1.03)	<0.001
MADV Ab absent, site ≠ Aruza†	591		*Ref*		
MADV Ab present, site ≠ Aruza†	10		5.7	(1.1–29.1)	0.038
MADV Ab absent, site = Aruza	140		0.6	(0.4–1.1)	0.114
MADV Ab present, site = Aruza	27		0.03	(0.0–0.2)	0.001
**Community**					
Tamarindo	176	8.0	0.1	(0.1–0.2)	<0.001
Pijivasal/Pirre 1&2	74	77.0	4.0	(1.9–8.3)	<0.001

*Adjusted for sex (OR = 1.4;*p* = 0.11); based on PRNT results

†This refers to sites other than Aruza, namely Tamarindo, El Real, Mercadeo, Pirre 1 & 2 and Pijivasal

Likelihood-ratio (LR) = 244.27; *df* = 13; *p* <0.001

Hosmer-Lemeshow χ^2^ = 12.81; *df* = 8; *p* = 0.12

## Discussion

Many features of MADV transmission remain unknown. Our findings of a lack of increasing seropositivity with age provides evidence that MADV has recently emerged, and characterizes activities and areas of exposure of MADV transmission as different from VEEV. This, in turn, suggested that there are different enzootic hosts and/or vectors for these otherwise similar viruses.

Examining the age structure of MADV and VEEV seroprevalences indicated substantial differences in the patterns of acquisition. Whereas VEEV showed a clear rise in the proportion of seropositive individuals with increasing age, suggesting continual and substantial exposure, MADV did not show any such trend (Fig 2). Additionally, there were substantial differences in the likelihood of infection in the same individuals implying a substantially different exposure history. These two points together provide compelling evidence that MADV did recently emerge in humans in this region. It remains unclear whether transmission has been sustained since the 2010 outbreak. Sampling in younger children is needed to evaluate this outcome.

The enzootic host(s) that maintain MADV in nature are not known, so examining the possible exposure sites and activities of daily life associated with MADV seropositivity could provide clues to identify them. Key activities that were positively correlated with human MADV antibodies were cattle ranching, farming and fishing. Cattle have infrequently been examined but have been noted to be rare incidental hosts for MADV [28,29]. Furthermore, blood meal analyses in Guatemala of the putative MADV vector, *Culex taeniopus*, identified cattle as the primary host[17]. Their potential role as amplifying hosts is wholly unknown. While cattle were not surveyed, rodents, opossums, bats, and birds were. The animal survey suggests a role for the agricultural and peri-domestic rodent species, the short-tailed cane rat and the black rat, respectively, as MADV hosts. The relatively high MADV seroprevalence in rodent species provides further evidence that rodents are likely to be the principal amplifying hosts as opposed to bats or birds. The short-tailed cane rat was found in high abundance in farms and pasture while the black rat was trapped in villages. These results corroborate our epidemiological findings reported showing associations of MADV seropositivity with farm, cattle/pasture, and the house.

The presence of VEEV antibodies was associated with farming and fishing, but also with activities in the forest. These findings are consistent with those reported on VEEV epidemiology in the Peruvian Amazon[30]. There, a positive association was seen between seropositivity and having a “high risk” occupation, defined as engaging in fishing, farming, logging, or exploration of petroleum in the rainforest. A study of the aquatic habitats of mosquitoes, including *Culex (Melanoconion) pedroi*, a VEEV vector in the Peruvian Amazon, characterized this species’ larval habitat to be small, shaded ground pools in the forest [31]. In addition, the forest-dwelling spiny rat (*Proechimys* spp.) has been implicated as an enzootic host of VEEV, further supporting our findings that forest exposure is a risk for VEEV infection[9]. Our small mammal survey also revealed high VEEV seroprevalence rates for the shrub and forest-dwelling rodents, the Bolivar rice rat (*Transandinomys bolivaris*) and the Tome’s spiny rat (*Proechimys semispinosus*), respectively.

We obtained evidence that the home is a site of exposure for both alphaviruses, as revealed by the significant associations of certain house features. Glazed windows and waste management were associated with reduced VEEV risk, while shrubs near the house were protective for MADV. Having glazed windows likely protects against the entry of mosquitoes (or could be a marker for social affluence), while municipal waste pick-up may diminish mosquito and/or host breeding or foraging. Similarly, having piped water may also reduce mosquito breeding by reducing standing water. Our findings of high black rat abundance in homes and black rat MADV seropositivity also suggested domiciliary transmission. Although village/urban MADV has not been described, urban VEEV was reported from Iquitos, Peru[7,30,32]. The possibility of urban VEEV transmission was further supported by experimental infections of *Aedes aegypti* from Iquitos with enzootic VEEV (subtypes ID, IIIC, IIID), revealing that this vector is moderately to highly susceptible to these viral strains[33].

There are additional factors operating at the level of the community or beyond that appear to influence alphavirus seroprevalence. This is borne out by the fact that our inclusion of the community as a variable remained significant in our multivariate analysis, and its effects could not be accounted for by the individual variables collected (such as activities, house structure, water and management). Host factors were important as well. There appears to be an interaction between MADV and VEEV seropositivity, in that exposure to one of the alphaviruses may be protective for the other, though this effect was only seen in Aruza. Such observations have been made in animal studies [5,34–36]. The IgG ELISA has extensive antibody cross-reactivity, and the effects of cross-protection as well as interference between these viruses has been observed in experimental vaccine studies[5,37]. Given the 16% seroprevalence of MADV and the 32% seroprevalence of VEEV in Aruza, one would expect to see 9 subjects with dual MADV and VEEV antibody presence. However, only 3 had dual seropositivity (*p* = 0.03). It is also conceivable that in some areas where MADV and VEEV transmission overlaps, a person could have become infected with both viruses concurrently, giving rise to the positive interaction seen here. This was observed in one patient during the 2010 outbreak[1]. These findings suggest that the VEEV seroprevalence in a population may alter the ability of MADV to emerge due to pre-existing cross-immunity.

Our study has several limitations. Due to the presumed long-term persistence of neutralizing VEEV and MADV antibodies, there may have been a significant time lapse between infection and our survey. For MADV, exposure was likely within 2–3 years of the survey, but for VEEV, infection may have occurred decades prior. People with VEEV antibodies who did not endorse risk factors such as farm, river, or forest exposures (n = 16) were significantly older (54.5 years, 95% CI 43.5–65.4) than the average participant age (27.7 years, 95% CI 26.2–29.2), indicating that these participants may have been exposed at a younger age and their current ecological conditions may have little bearing on their original risk for VEEV. Also, antibody titers may wane over time, leading to underestimates of prior exposure in older age groups. Another limitation is posed by the heterogeneous nature of this region and the study villages. The ethnic makeup of the population varies from indigenous (Embera-Wounaan, Kuna tribes) to mestizo to people of African descent. Data on ethnicity was not gathered; there could be a difference in susceptibility or exposure to MADV and/or VEEV according to ethnic group. An additional limitation is that our study examined the associations of personal and household risk factors and seropositivity to MADV and VEEV, but did not consider broader contextual factors adjacent to the current household such as land use, migration policies, and community-level use of waste and water management systems. Such analyses are currently underway. Model selection here was undertaken using the purposeful selection method described by Hosmer and Lemeshow, in which variables are examined and added according to significance and relevance in a manual stepwise fashion[38]. The resulting models are meant to provide the most useful insights with regards to risk factors associated with each disease.

**Fig 4:**
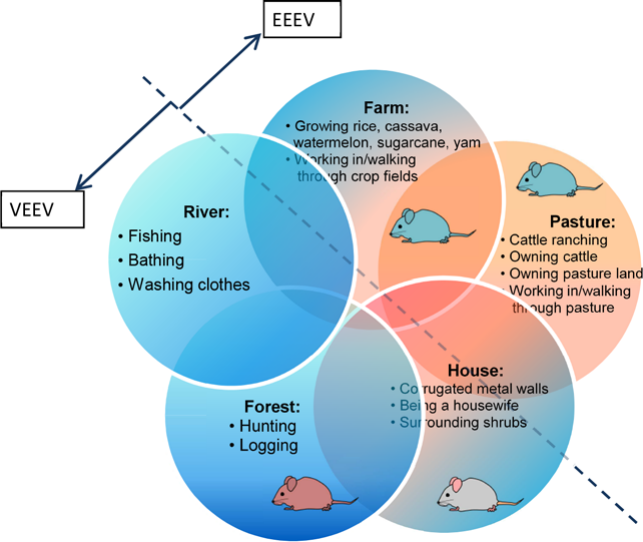
VEEV and MADV risk factors according to the sphere of activity. Blue mice represent *Z*. *brevicauda*, the gray mouse *R*. *rattus*, and the ruddy mouse *T*. *bolivaris*.

In summary, human exposure to MADV varies by community, appears to have emerged recently, and has an overlapping but distinct set of risk factors compared to VEEV. VEEV and MADV antibody presence is associated with farming, fishing, and house features likely linked to mosquito breeding and entry. Whereas VEEV seropositivity is also related to forest exposure, MADV seropositivity is associated with cattle ranching. These risk factors provide some insight into possible similarities and differences in the enzootic transmission cycles for these two sympatric viruses (Fig 4). The additional finding that prior alphavirus exposure may protect against future alphaviral infection raises the possibility that cross-immunity may influence disease emergence. To further define MADV and VEEV transmission factors and epidemiology, vector studies (including blood meal analyses, infection rates and ecology), experimental infections of putative hosts, and studies on land use and land cover are needed.

## Supporting Information

S1 Table MADV and VEEV plaque reduction neutralization test titers for animal samples. (XLS)

S2 Table MADV and VEEV plaque reduction neutralization test titers for human subjects. (XLS)

S3 Table Marginal effects of VEEV antibodies by community. (DOCX)

S4 Table Marginal effects of MADV antibodies by community. (DOCX)

S5 Table Human serosurveillance variables and interaction terms. (DOCX)
