# Supplementary material for: Epidemiology of Emergent Madariaga Encephalitis in a Region with Endemic Venezuelan Equine Encephalitis: Initial Host Studies and Human Cross-Sectional Study in Darien, Panama
Source: PLoS Negl Trop Dis. 2016 Apr 21;10(4):e0004554. doi: 10.1371/journal.pntd.0004554 (PMC4839771; doi:10.1371/journal.pntd.0004554)
Supplement: S3 Table — (DOCX) [file pntd.0004554.s003.docx]

**Table S3. Marginal effects of VEEV antibodies by community, expressed as the change in probability of MADV seropositivity, holding all other variables at their mean values**

| **Interaction term** | Marginal effect | Standard error (delta method) | P>\|z\| |
| --- | --- | --- | --- |
| Site ≠ Aruza | 0.017 | 0.010 | 0.087 |
| Site = Aruza | -0.126 | 0.040 | 0.001 |
