# Supplementary material for: Epidemiology of Emergent Madariaga Encephalitis in a Region with Endemic Venezuelan Equine Encephalitis: Initial Host Studies and Human Cross-Sectional Study in Darien, Panama
Source: PLoS Negl Trop Dis. 2016 Apr 21;10(4):e0004554. doi: 10.1371/journal.pntd.0004554 (PMC4839771; doi:10.1371/journal.pntd.0004554)
Supplement: S4 Table — (DOCX) [file pntd.0004554.s004.docx]

**Table S4. Marginal effects of MADV antibodies by community, expressed as the change in the probability of VEEV seropositivity holding other model variables at their mean values**

| **Interaction term** | Marginal effect | Standard error (delta method) | P>\|z\| |
| --- | --- | --- | --- |
| Site ≠ Aruza | 0.408 | 0.179 | 0.022 |
| Site = Aruza | -0.156 | 0.043 | <0.001 |
