# Supplementary material for: Epidemiology of Emergent Madariaga Encephalitis in a Region with Endemic Venezuelan Equine Encephalitis: Initial Host Studies and Human Cross-Sectional Study in Darien, Panama
Source: PLoS Negl Trop Dis. 2016 Apr 21;10(4):e0004554. doi: 10.1371/journal.pntd.0004554 (PMC4839771; doi:10.1371/journal.pntd.0004554)
Supplement: S5 Table — (DOCX) [file pntd.0004554.s005.docx]

**S5. Human serosurveillance variables:**

| **Variable** | **Description** |
| --- | --- |
| Age | In years |
| Sex | Male/female |
| Site | Aruza, Tamarindo, El Real de Santa Maria, Mercadeo, Pirre I/II & Pijivasal |
| Location | House GPS coordinates |
| Residency (months vs years) | Dichotomous: 3 – 11 months; 1 year or more |
| Lived in different province? | Yes/no |
| Occupation | Professional; technician; office worker; day laborer; house keeper; student; unemployed; farmer; pre-school; cattle rancher; other |
| Agricultural activities | Number of hours per week on average during the past year engaged in the following categories:  Farming  Domestic animal rearing  Chicken rearing  Cattle rearing  Hunting  Fishing  Other  None |
| Farming | Crops grown:  Melon  Corn  Rice  Sugarcane  Legumes  Watermelon  Sorghum  Pasture  Yuca  Plantain  Other  None |
| Activities | Frequency (always, sometimes, never) of the following categories:  Walking through pasture  Walking through crops  Playing in pasture  Playing in crops  Clearing shrub  Working in pasture  Working in grain deposits  Working in lumber mills  Working in the forest  Working in chicken coups  Working in pig farms  Working in abattoirs  Cleaning cabins  Handling animal feed  Washing clothes in the river  Bathing in the river  Bathing in ponds |
| House construction | |
| Floor construction | Categorical -  Dirt  Wood  Tile  Ceramic  Cement  Mosaic |
| Wall construction | Categorical –  Wood  Adobe  Wattle and daub  Cement  Corrugated metal  Mud |
| Window construction | Categorical –  Ornamental  Wood  Metal  Glass  Sliding |
| Roof construction | Categorical –  Tile  Corrugated metal  Straw  Palm |
| Presence of domestic animals in/near the home | Dichotomous –  Cats  Dogs  Birds  Goat  Pigs  Cattle  Horses |
| Insecticide applied to walls | Yes/no |
| Peri-domiciliary vegetation and animals | Shrubs around the house (if yes, distance in m)  Crops around the house (if yes, distance in m)  Pig pen around the house (if yes, distance in m)  Chicken coup around the house (if yes, distance in m) |
| Waste management | Dichotomous -  Food waste around the house |
|  | Trash around the house |
|  | Latrine |
|  | Sink |
|  | Septic tank |
|  | Connected to municipal sewage/water supply |
|  | Disposal of waste water outside on the patio |
|  | Disposal of waste water in a sink |
| Solid waste disposal | Dichotomous –  Bury waste  Burn waste  Throw into river  Throw into lake/ocean  Municipal waste service  Accumulate in a pile |
| Water source | Dichotomous –  Piped water  Well water  River water  Rain water |
| Socioeconomic status | Dichotomous –  Electricity  Metered electricity  Radio  TV  Cable TV  Telephone  Cell phones |
| Income | Balboa/year per household |

**Interaction terms:**

MADV Model

| Interaction terms | Description |
| --- | --- |
| Cattle ranching*Farm exposure | Too few observations when cattle ranching=1 and farm exposure=0 |
| Cattle ranching*Fishing | Too few observations when cattle ranching=1 and fishing=1 |
| Farm exposure*Fishing | Too few observations when farm exposure=0 and fishing=1 |
| Cattle ranching*shrub within 10m | Too few observations when cattle ranching=1 and shrub within 10m=1 |
| Farm exposure*shrub within 10m | AIC=0.315 vs AIC=0.314 for main effects model; rejected |
| Fishing*shrub within 10m | Interaction term not significant |
| Cattle ranching*site | Not enough observations |
| Farm exposure*site | Non convergence of model |
| Fishing*site | Non convergence of model |
| Shrub within 10m*site | AIC=0.387 vs AIC=0.314 for main effects model; rejected |
| Cattle ranching*VEEV | Not enough observations |
| Farm exposure*VEEV | Interaction term nonsignificant (p=0.683) |
| Fishing*VEEV | AIC=0.313 vs AIC=0.314 for main effects model; interaction term and “fishing” are highly collinear |
| Shrub within 10m*VEEV | Interaction term nonsignificant (p=0.277) |
| VEEV*site | AIC=0.294 vs AIC=0.314 for main effects model; interaction term not collinear 🡪 term included in model |

**Interaction terms:**

VEEV Model

| Farming*working in the forest | Terms are collinear |
| --- | --- |
| Farming*fishing | Too few observations |
| Working in the forest*fishing | Too few observations |
| Farming*glazed windows | Too few observations |
| Working in the forest*glazed windows | Too few observations |
| Fishing*glazed windows | Too few observations |
| Farming*municipal waste pick up | Interaction term nonsignificant (p=0.138) |
| Working in the forest*municipal waste pick up | Too few observations |
| Fishing*municipal waste pick up | Too few observations |
| Farming*piped water | Interaction term nonsignificant (p=0.165) |
| Working in the forest*piped water | Interaction term nonsignificant (p=0.118) |
| Fishing*piped water | AIC=0.979 vs AIC=0.977 for main effects model; rejected |
| Glazed windows*municipal waste pick up | AIC=0.978 vs AIC=0.977 for main effects model; rejected |
| Glazed windows*piped water | AIC=0.978 vs AIC=0.977 for main effects model; rejected |
| Municipal waste pick up*piped water | Interaction term nonsignificant (p=0.848) |
| Farming*age | Interaction term nonsignificant (p=0.395) |
| Working in the forest*age | Interaction term nonsignificant (p=0.537) |
| Fishing*age | Interaction terms are collinear |
| Glazed windows*age | Interaction term nonsignificant (p=0.357) |
| Municipal waste pick up*age | Interaction term nonsignificant (p=0.978) |
| Piped water*age | Interaction term nonsignificant (p=0.289) |
| Farming*MADV | Interaction term nonsignificant (p=0.913) |
| Working in the forest*MADV | Too few observations |
| Fishing*MADV | Too few observations |
| Glazed windows*MADV | Too few observations |
| Municipal waste pick up*MADV | Too few observations |
| Piped water*MADV | Interaction term nonsignificant (p=0.763) |
| Age*MADV | Interaction term nonsignificant (p=0.695) |
| Farming*site | AIC=0.977 vs AIC=0.977 for main effects model; rejected |
| Working in the forest*site | Interaction terms nonsignificant (p=0.575) |
| Fishing*site | Too few observations |
| Glazed windows*site | Too few observations |
| Municipal waste pick up*site | Too few observations |
| Piped water*site | Too few observations |
| Age*site | Interaction terms nonsignificant (p=0.706) |
| MADV*site | AIC=0.973 vs AIC=0.977 for main effects model; terms not collinear 🡪 term included in the model |
